# Supplementary figures and images for: Membrane curvature sensing and symmetry breaking of the M2 proton channel from Influenza A
Source: eLife. 2024 Aug 16;13:e81571. doi: 10.7554/eLife.81571 (PMC11383528; doi:10.7554/eLife.81571)

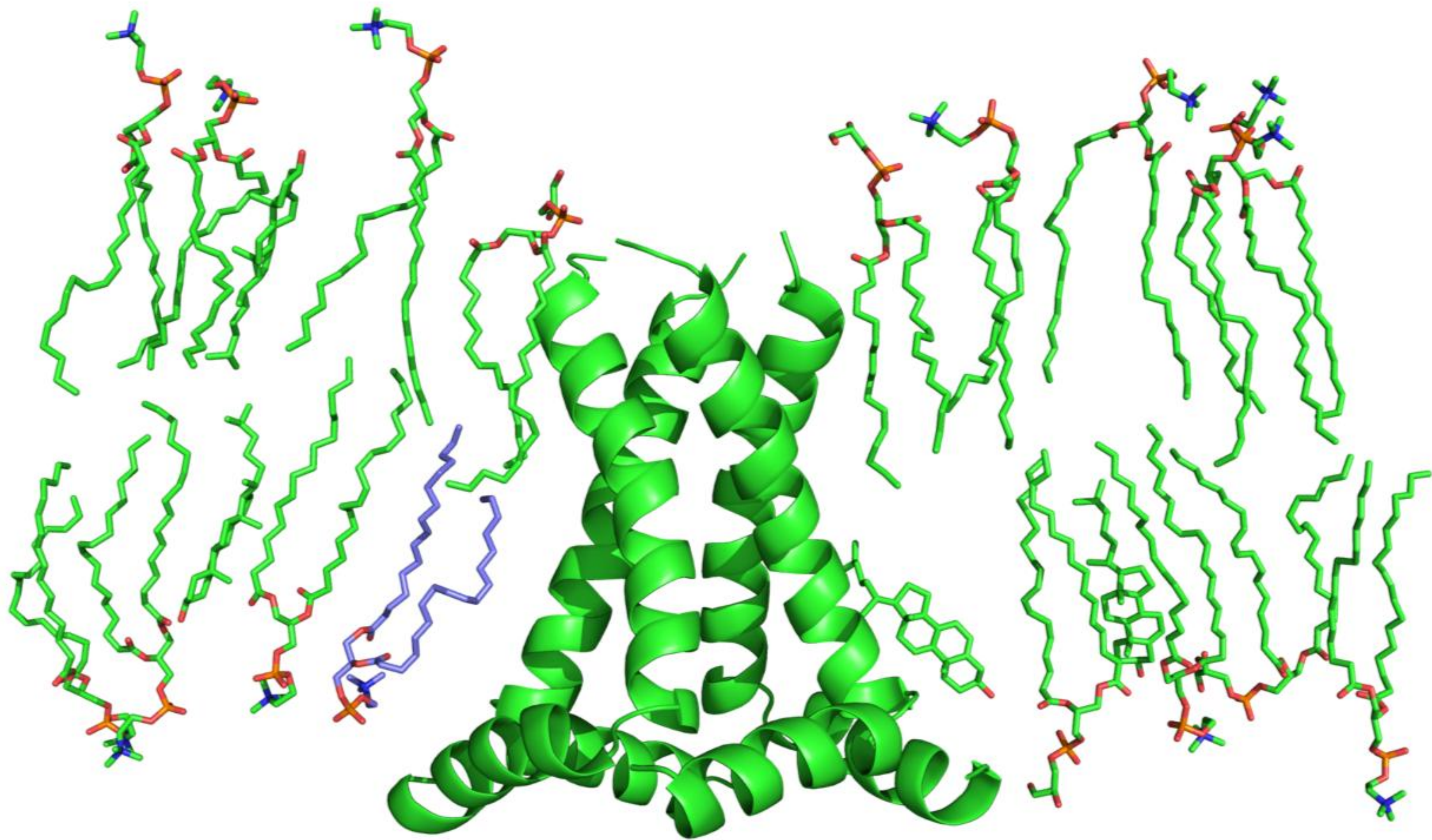

Supplement: Figure 7—source data 1. [file elife-81571-fig7-data1.pdf]

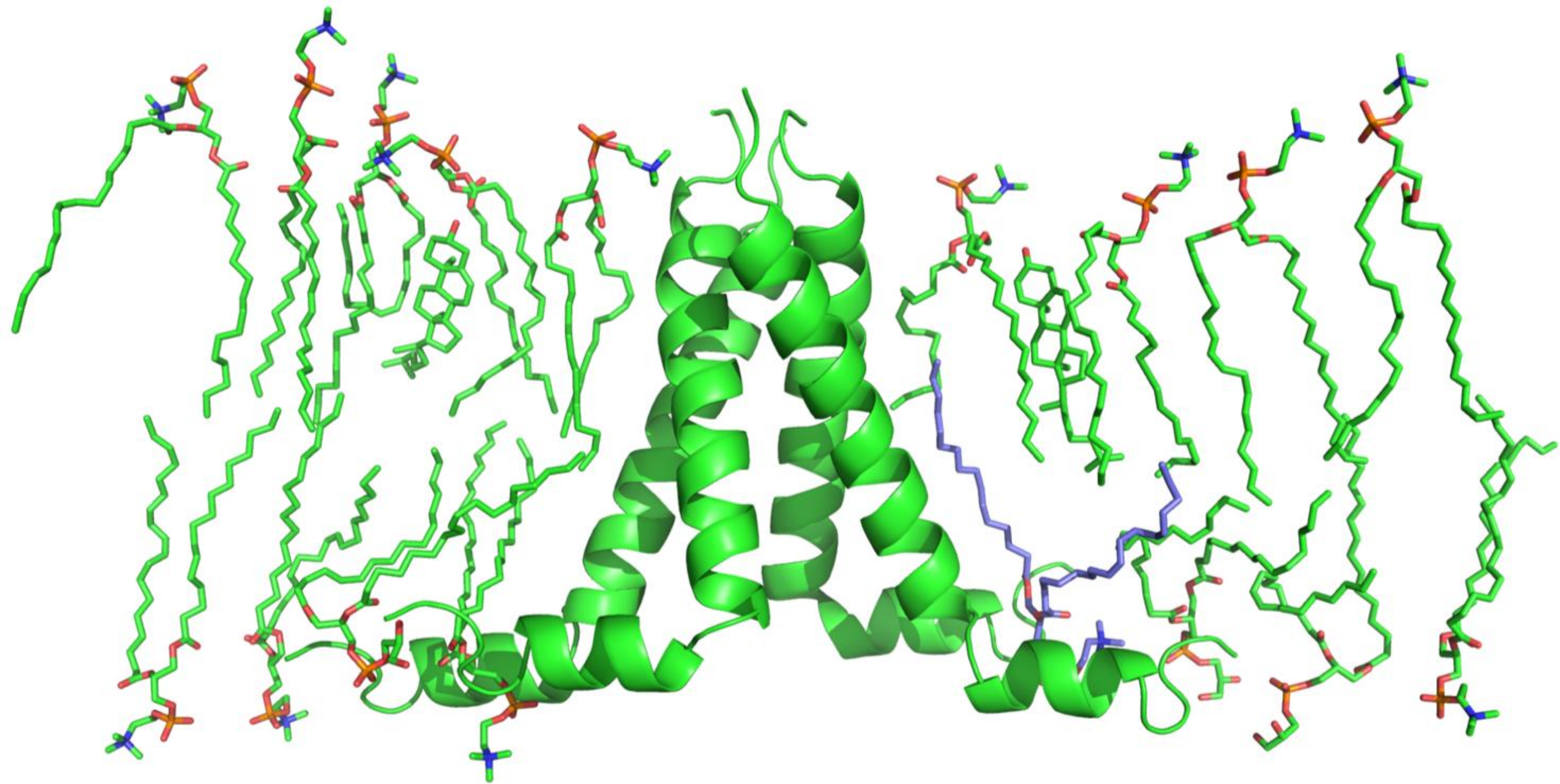

Supplement: Figure 7—source data 2. [file elife-81571-fig7-data2.pdf]

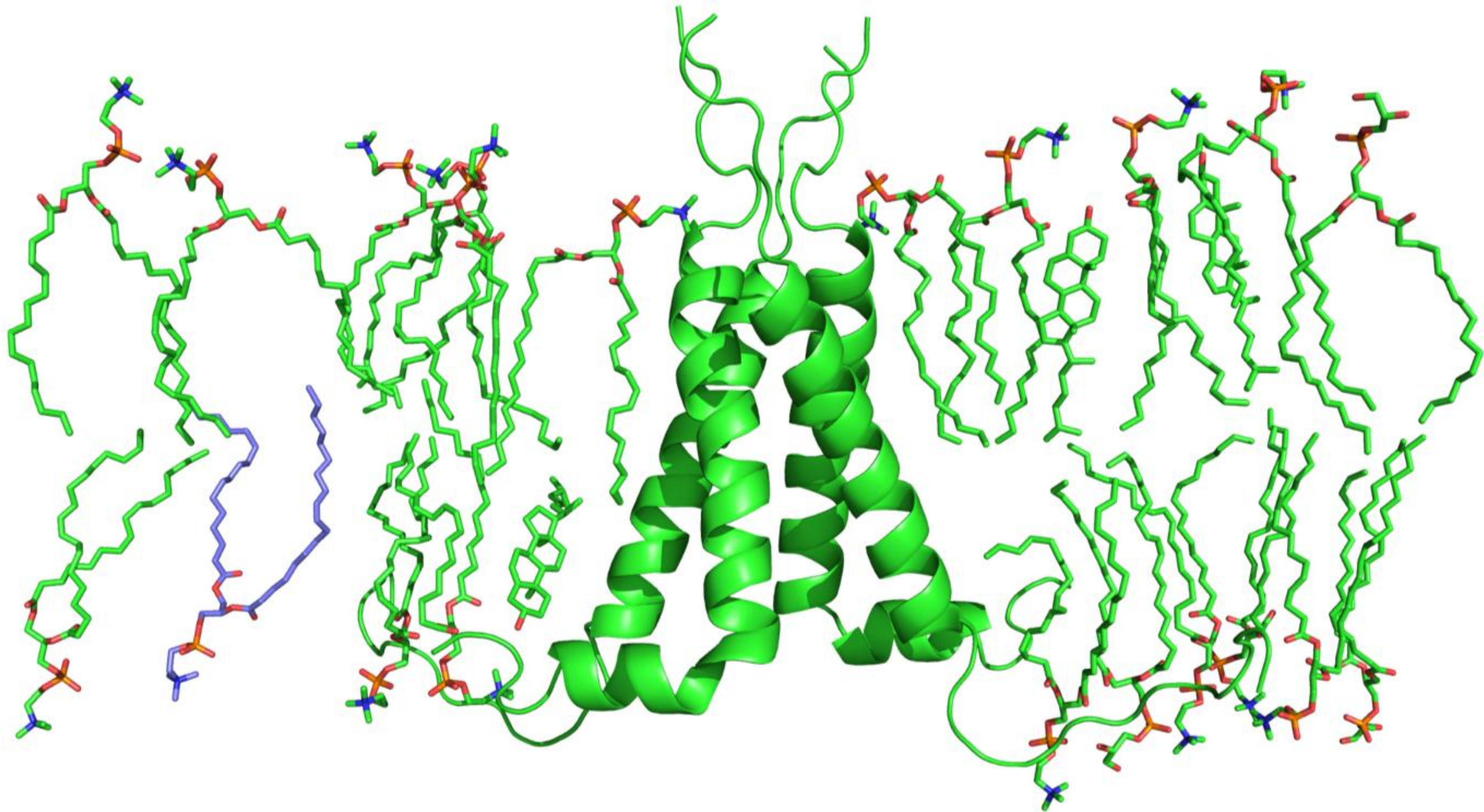

Supplement: Figure 7—source data 3. [file elife-81571-fig7-data3.pdf]
